# Hooded crows (*Corvus cornix*) manufacture objects relative to a mental template

**DOI:** 10.1007/s10071-024-01874-6

**Published:** 2024-04-29

**Authors:** Anna A. Smirnova, Leia R. Bulgakova, Maria A. Cheplakova, Sarah A. Jelbert

**Affiliations:** 1 Faculty of Biology, Lomonosov Moscow State University, Leninsky Gory, 1, 12, Moscow, 119899 Russia; 2 School of Psychological Science, University of Bristol, Priory Road, Bristol, BS8 1TU UK

**Keywords:** Tool manufacture, Template matching, Representations, Emulation, Hooded crows

## Abstract

It was recently found that not only tool-specialized New Caledonian crows, but also Goffin cockatoos can manufacture physical objects in accordance with a mental template. That is, they can emulate features of existing objects when they manufacture new items. Both species spontaneously ripped pieces of card into large strips if they had previously learned that a large template was rewarded, and small strips when they previously learned that a small template was rewarded. Among New Caledonian crows, this cognitive ability was suggested as a potential mechanism underlying the transmission of natural tool designs. Here, we tested for the same ability in another non-specialised tool user–Hooded crows (*Corvus cornix*). Crows were exposed to pre-made template objects, varying first in colour and then in size, and were rewarded only if they chose pre-made objects that matched the template. In subsequent tests, birds were given the opportunity to manufacture versions of these objects. All three crows ripped paper pieces from the same colour material as the rewarded template, and, crucially, also manufactured objects that were more similar in size to previously rewarded, than unrewarded, templates, despite the birds being rewarded at random in both tests. Therefore, we found the ability to manufacture physical objects relative to a mental template in yet another bird species not specialized in using or making foraging tools in the wild, but with a high level of brain and cognitive development.

**Supplementary Information:**

The online version contains supplementary material available at 10.1007/s10071-024-01874-6.

## Introduction

New Caledonian crows (*Corvus moneduloides*) are well known for their impressive tool-related behaviours. All members of this species routinely manufacture tools for extractive foraging in the wild (including straight sticks, hooked twigs and tools torn from the leaves of the Pandanus tree), with some of these tools requiring complex manufacture (Hunt 1996; 2000a, b; Hunt and Gray 2002; 2004a, b; Rutz et al. 2010; Troscianko and Rutz 2015; St Clair et al. 2018).

Their natural tool use behaviour likely results from a complex interplay of innate components with different types of learning and, possibly, involves the contribution of higher cognitive processes (Kenward et al. 2006; Bluff et al. 2007).

The contribution of the innate component is evidenced, for example, by the fact that young birds, raised in isolation, develop basic stick tool manufacture spontaneously without any social input (Kenward et al. 2005, 2006; Hunt et al. 2007). However, they do not manufacture the more complex tool designs observed in the wild.

Under experimental conditions, New Caledonian crows have demonstrated sophisticated cognitive abilities, including the ability to manufacture tools from novel materials, select or manufacture a tool depending on the specifics of the task, create multi-component tools, use one tool to access another tool to access food, preplan their behaviours into the future while using tools, infer the properties of objects, and attend to causal regularities (Chappell and Kacelnik 2002; 2004; Weir et al. 2002; Weir and Kacelnik 2006; Bluff et al. 2007; Taylor et al. 2007; 2009a, 2009b; Laumer et al. 2017; von Bayern et al. 2018; Jelbert et al. 2019; Gruber et al. 2019; Boeckle et al. 2020). These findings implicitly suggest that higher cognitive processes (such as reasoning, causal understanding or logical inference) may contribute to their natural tool-using behaviour.

Recently, it has been demonstrated that New Caledonian crows have the capacity for mental template matching–a form of manufacture by emulation–which could potentially enable the cultural transmission of different tool designs in the wild (Jelbert et al. 2018; Taylor and Jelbert 2020). The existence of cultural traditions has been suggested from the observation that the shape of New Caledonian crow pandanus tools varies across populations, with specific tool designs having no obvious ecological correlates, and persisting for several decades (Hunt 2000a; Hunt and Gray 2003; Hunt et al. 2006; Hunt and Uomini 2016). This distribution of simpler and more complex designs provides tentative evidence that these designs have not emerged independently, but rather have gone through a process of cumulative change. However, we have yet to find convincing evidence of behavioural imitation (Logan et al. 2016), typically thought necessary for high-fidelity transmission of information.

In the wild, New Caledonian crows can feed their young for up to 2 years (Holzhaider et al. 2011), which is one of the longest-known periods of regular extended parental feeding among birds (Hunt et al. 2012). During this time juvenile New Caledonian crows are often in close proximity to their tool-making parents and will often use their parents’ discarded tools for foraging, providing opportunities for social transmission of information (Bluff et al. 2007; Holzhaider et al. 2010a, b; Hunt et al 2012).

Social learning could occur not only by observing how other crows make tools but also by observing the end-products, a form of emulation. By inspecting abandoned tools, New Caledonian crows could perhaps form an idea of what a manufactured tool should look like (i.e. form a mental template of a particular tool design), and then reproduce this design in their own manufacture (Bluff et al. 2007; Holzhaider et al. 2010a). In favour of this hypothesis is, for example, the fact that of the four crows raised in isolation, functional tools were made from pandanus leaves not only by birds that had been shown how to do so but also by a crow that had been provided with pre-made tools (Kenward et al. 2006).

To test whether mental template matching could be a plausible mechanism underpinning NC crow tool-making traditions Jelbert et al. (2018), as mentioned above, assessed the ability of New Caledonian crows to manufacture novel objects that matched a mental template (or a representation) of a previously rewarded stimulus. Eight New Caledonian crows were first trained to drop ready-made squares of white paper into a vending machine to receive rewards. They then received a spontaneous manufacture test in which only very large sheets of white paper were provided. Half of the birds ripped sections from these sheets without training and dropped them into the vending machine to obtain rewards. The other half of the birds were trained to rip paper.

Then the birds were presented with ready-made card of two different colours and were rewarded only by choosing the objects of one colour. 6 of 8 birds learned to drop only a rewarded colour of paper into the vending machine within 30 training blocks. In the following Color test, these 6 birds were not given ready-made objects to choose from but were provided with very large sheets of both the rewarded and unrewarded colours from which the birds could tear sections (i.e. manufacture their own card pieces) to insert into a vending machine to obtain rewards. In this test, subjects were rewarded for inserting pieces from the correct colour only. 5 of 6 birds ripped pieces of paper from the sheet of the previously rewarded colour in at least 19 of 24 trials.

The next step examined whether birds were capable of flexibly recalling and reproducing the size of previously reinforced templates. The birds were presented with ready-made pieces of card of two different sizes (large: 60 × 40 mm and small: 25 × 15 mm rectangles) and learned that only one size was rewarded. In the Size test birds were provided with two very large sheets of card only. After manufacturing 20 pieces, they were trained that the pre-made pieces of paper of alternative size were rewarded, and a second Size test was conducted. To prevent trial-and-error learning, in both Size tests, the crows were rewarded at random on 50% of the trials, regardless of the size of the ripped pieces that they inserted. In the Size tests, six of the eight birds manufactured smaller pieces when they had previously learned that small templates were rewarded and larger pieces when large templates were previously rewarded. The two birds that did not were both juveniles. Here, the adult birds manufactured items that matched the relative size of the previously rewarded templates without being rewarded for doing so during manufacture test trials and without templates being present at the time of manufacture. One bird showed tentative evidence of matching the absolute size of the template, making secondary modifications to both large and small templates to adjust their size. Thus, mental template matching emerges as a plausible mechanism by which New Caledonian crow tool designs could be transmitted across birds.

More recently, the capacity for mental template matching has been studied among another species, Goffin's cockatoos (*Cacatua goffiniana*) (Laumer et al. 2021). Unlike New Caledonian crows, Goffin's cockatoos are not specialised tool users. However, it has recently been discovered that some individuals do use tools in their natural habitat (Osuna-Mascaró and Auersperg 2018; O’Hara et al. 2021). Following a single observation of tool-related foraging behaviour (Osuna-Mascaró and Auersperg 2018), O’Hara et al. (2021) observed two short-term captive but otherwise wild cockatoos, using a set of up to three different tools specifically crafted out of wood for three different functions (a sturdy tool for wedging, a slim tool for cutting, and a long, broad tool for spooning) to access the seed content of a fruit stone (O’Hara et al. 2021).

In the laboratory, these birds have shown the capacity for flexible tool use and manufacture (Auersperg et al. 2012, 2014, 2017; Lambert et al. 2015, 2019; Beinhauer et al. 2019; Habl and Auersperg 2017; Laumer et al. 2016, 2017; 2021; Osuna-Mascaró et al. 2022; 2023). For example, they manufactured stick-type tools across three different materials, with each material requiring different manipulation patterns (Auersperg et al. 2016) and can modify the specific feature (length) of the manufactured tools depending on the specific task demands (Auersperg et al. 2018).

The capacity for flexible tool use has also been found in some members of the corvid family that are not specialised tool users. For example, northern blue jays (*Cyanocitta cristata*) have torn pieces from pages of newspaper and used them as tools to obtain food out of reach (Jones and Kamil 1973). Rooks (*Corvus frugile*gus) made hook tools from pieces of straight wire to extract bait (Bird and Emery 2009) and were able to solve the modified trap-tube task, which may indicate the use of causally relevant functional information (Seed et al. 2006). Ravens are capable of planning for tool use (throwing a rock into a transparent tube to get bait off a rocking platform) and bartering with delays of up to 17 h (Kabadayi and Osvath 2017). Although ravens' tool use in the wild may be more common than widely perceived (Jacobs and Osvath 2023). Thus, non-tool-using species can often display comparable cognitive abilities to tool-users on tool-related tasks. In the meantime, tool-using species sometimes do not outperform their non-tool-using relatives on physical cognition tests (Teschke et al. 2011, 2013).

This also applies to the ability to form mental representations. Parrots and crows can extract relations among items and between relations, form abstract categories not tied to specific perceptual features and use abstract representations (Pepperberg 1987; 2013; 2018; 2020; Wilson et al. 1985; Seed et al. 2009; Lazareva et al. 2004; Wright et al. 2016; 2017; Magnotti et al. 2015; 2017; Smirnova et al. 2000; 2015; 2021; Obozova et al. 2015; Balakhonov and Rose 2017; Samuleeva and Smirnova 2020).

Our investigation aimed to find out whether Hooded crows (*Corvus cornix*) could also produce objects according to a mental template. Hooded crows, like Goffin's cockatoos, are not specialised tool users. Like many other members of the corvid family, Hooded crows drop mussels onto rocks, which is a typical example of proto-tool use, and drop pieces of branches onto intruders during the nesting season (Shumaker et al. 2011; Davenport et al. 2014), although the latter may be due to a displacement behaviour that is typical for them when they are frustrated (Heinrich 1988).

We previously revealed the ability of some Hooded crows to solve sophisticated variants of string-pulling tasks (Bagotskaya et al. 2012). Here, two of the four crows chose the correct string more often in a slanted parallel string task, where the bait was closest to the far end of an "empty" string. Similarly, four crows out of eight passed a task requiring them to pull on one angled baited string and ignore a straight "empty" string. Six of the eight crows solved a task with two baited strings, where one was broken and one was intact. However, it remains unclear whether crows understand the physical principles underlying a string-pulling task or whether this result is due to rapid associative learning by using perceptual-motor feedback (Taylor et al. 2010, 2012).

In the present work we investigated whether the capacity for mental template matching was shared among corvids or limited to New Caledonian crows. To maintain comparability between species we used the same paradigm as Jelbert et al. (2018) and Laumer et al. (2021) with slight modifications: not only in the Size test but also in the Colour test the crows were randomly rewarded to prevent trial-and-error learning. The Colour test was conducted to investigate whether the crows would manufacture paper pieces out of the same colour as previously rewarded templates. In the Size test, we tested whether the crow would manufacture big or small paper pieces, depending on the previously rewarded template.

## Methods

### Subjects, housing and experimental history

Three adult Hooded crows (*Corvus cornix*) were tested. All of them were rescued from the wild due to injuries and were housed in the outdoor group aviary (500 × 250 × 300 cm) on the territory of the Lomonosov Moscow State University Botanical Garden. The aviaries are equipped with perches (tree branches, wooden ladders, and stumps), wooden shelters, toys, metal and ceramic feeders and plastic basins with water.

The birds are kept on an ad libitum diet (rat and mouse carcasses, steamed crops and seeds with added vegetable oil and vitamins, eggs, seasonal fruits and vegetables, and fresh water). If the crows refused to work in the experiment, then they received food without animal protein for 1 or 2 days. The experiments were conducted from 2022 to 2023. For the study, birds were transferred to an experimental room (250 × 400 cm).

Glaz was kept for over 15 years, and Rodya and Joe for four. The sex of the birds is not known exactly. Based on sex-size dimorphism and behaviour, it can be assumed that they are males.

Prior to this experiment, all birds had participated in Aesop’s fable and string-pulling experiments. One of them (Glaz) had acquired an abstract sameness rule after mastering a series of highly varied identity matching-to-sample tasks and later spontaneously applied this rule to perform relational matching-to-sample tasks (Smirnova et al. 2015).

All experiments were appetitive, non-invasive and based exclusively on behavioural tests. They were conducted in full compliance with the bioethical requirements of Directive 2010/63/EU and Federal Law of 27.12.2018 N498-FZ (ed. of 27.12.2019) "On Responsible Treatment of Animals and Amendments to Certain Legislative Acts of the Russian Federation". All applicable international, national and institutional guidelines for the care and use of animals were followed. The studies have been approved by the MSU Bioethics Commission meeting № 157-d.

### Experimental setup

**Fig. 1:**
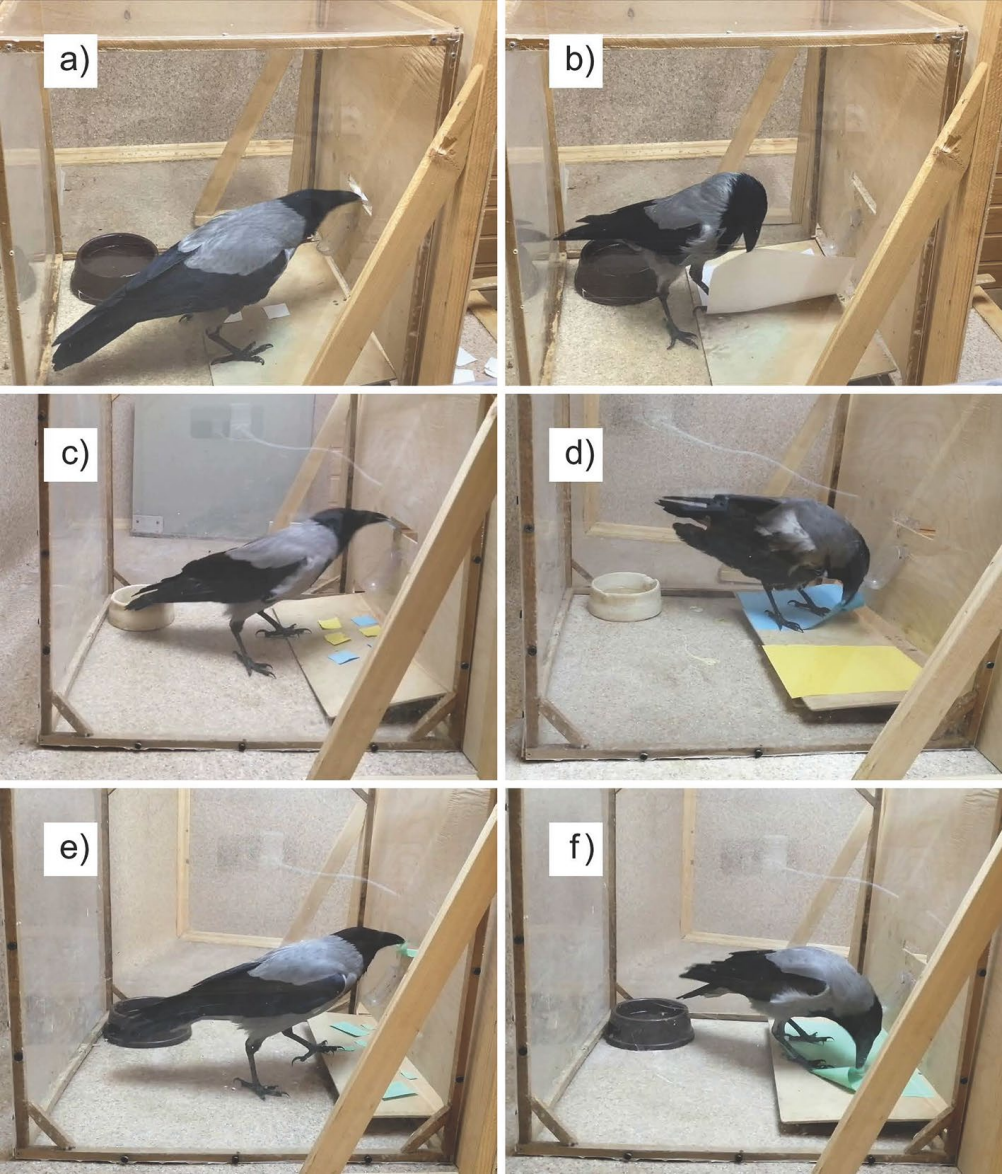
**a** Pre-training. **b** Spontaneous manufacture test. **c** Colour learning. **d** Colour test. **e** Size learning. **f** Size test

For the experiment, subjects were placed in a transparent plexiglass cage (50 × 50 × 50 cm) without a front wall. Instead of the missing wall, a plywood screen was placed at the cage, behind which the experimenter was located. The screen had three holes (Fig. 1). At the bottom of the screen, there was a slit (45 × 3 cm) through which a wooden tray (24 × 40 cm) with items was pushed into the cage. At the top, there was a smaller slit (12 × 1.5 cm) into which the crow could insert items. The third hole was made between the upper and lower slits, beneath which a feeder was placed on the bird's side. The experimenter placed a reinforcement–a mealworm larva – in this hole. The cage was equipped with a bowl of water.

### Experimental procedures

#### Pre-training

Subjects were first trained to drop small stones, then white paper squares (35 × 35 mm; hereinafter 80 GSM), into the upper slit. At first, the experimenter placed the stone into the upper slit themselves. When the stone fell toward the experimenter for any reason, the experimenter rewarded the birds with a mealworm larva through the middle hole with a feeder. When subjects purposefully pushed the stone in the direction of the experimenter 10 times, a tray with 8 stones was put into the cage through the bottom slit. The birds then received a reward when it picked up the stone from the tray by itself and placed it into the slit. When the birds placed all 8 stones into the slit in three consecutive presentations of the tray, a tray with 8 pieces of white paper (35 × 35 mm) was put into the cage (Fig. 1a). Subjects received a reward when they placed the piece of paper into the slit. The next stage was carried out when the bird placed all 8 pieces into the slit in three consecutive presentations of the tray. During all phases, the experimenter sat behind a plywood screen and could see the crow only if they intentionally looked into the upper slit. They didn't move or look at the crow when the bird made its choice.

### Spontaneous manufacture test

The crow's ability to independently manufacture pieces of paper that could be placed into the upper slit for a reward was evaluated. At the beginning of each day, the bird received a reminder trial – presentation of a tray with 8 pieces of white paper (35 × 35 mm) on it. The test trial was carried out only if the bird put all eight pieces of white paper into the slit. Otherwise, the entire procedure was repeated on another day. In the test trial, a tray with a white sheet of A4 paper was put into the cage (Fig. 1b). On each day of the experiment, there were three presentations of a tray, each lasting 15 min. If the birds started tearing off pieces of paper and placing them into the slit by itself, then the next stage was carried out after at least 24 pieces were made. All manufactured items dropped into the slit were rewarded.

### Colour learning

The birds were then trained that only inserting items of a certain colour was rewarded. A tray with 8 pieces (35 × 35 mm) of paper was put into the cage: 4 blue and 4 yellow pieces. The pieces of different colours were placed on the tray semi-randomly. The crows received a reward for placing pieces of only blue colour into the slit (Fig. 1c). The crows received a varying amount of trials (presentations of the tray) per day depending on their individual motivation (usually around 30 trials). The next stage (colour test) was carried out when, in three consecutive trials (presentations of the tray), the bird put all 4 pieces of the blue colour and no yellow pieces into the slit (movie is given in Online Resource 1).

### Colour test

The Colour test was conducted to investigate whether the crows would manufacture paper pieces out of the same colour as previously rewarded items. At the beginning of each day, the bird received a reminder trial–a presentation of a tray with 4 blue and 4 yellow pieces (35 × 35 mm) on it. The test trial was carried out only if the bird put all 4 pieces of the blue colour and no yellow pieces into the slit. Otherwise, the entire procedure was repeated on another day. In the test trial, a tray was put into the cage, with two sheets of paper (A5) of two colours (the same as for the previous stage–blue and yellow) placed on both sides of the tray (Fig. 1d). The sheets were placed on the different sides of the tray semi-randomly. To prevent trial-and-error learning during the test, the birds were randomly rewarded for 50% of the items they manufactured and placed into the slit, regardless of the colour. After the bird had torn off 3–4 pieces of paper, the tray with the sheet was slowly moved out of the cage, completing the trial. As a result, crows would tear off up to 6 pieces per trial. The test was completed when the bird had torn off and placed at least 24 pieces in the slit. If a crow did not manufacture a piece of paper within 15 min, the entire procedure was repeated on the subsequent testing day (movie is given in Online Resource 1).

### First size learning

The birds were trained that only inserting items of a certain size was rewarded. A tray with 8 pieces of paper was put into the cage: 4 large pieces (40 × 60 mm) and 4 small pieces (15 × 25 mm). The pieces were all the same colour (red for Glaz and Joe, orange for Rodya). The pieces of different sizes were placed semi-randomly (Fig. 1e). The crows were rewarded for placing only pieces of one size into the slit: Glaz and Joe were rewarded for small pieces, Rodya –for big ones. The crows received a varying amount of trials per day depending on their individual motivation (usually around 30 trials). The next stage was carried out when, in three consecutive presentations of the tray, the birds put 4 pieces of the rewarded size and none of the other size into the slit (movie is given in Online Resource 1).

### First size test

The size test was conducted to investigate whether the crows would manufacture paper pieces similar in size to previously rewarded templates. At the beginning of each day, the bird received a reminder trial–a presentation of a tray with 4 large (40 × 60 mm) and 4 small pieces (15 × 25 mm). The test trial was carried out only if the bird put all 4 pieces of the previously rewarded size and no pieces of other size into the slit. Otherwise, the entire procedure was repeated on another day. In the test trial, a tray with an A4 sheet of paper was put into the cage (Fig. 1f). To prevent trial-and-error learning during the test, the birds were randomly rewarded for 50% of the items they manufactured and placed into the slit, regardless of the size. The test was completed when the bird made and placed at least 24 pieces. If crow did not manufacture a piece of paper within 15 min, the entire procedure was repeated on the subsequent testing day (movie is given in Online Resource 1).

### Reversal size learning

To further explore whether crows would spontaneously manufacture larger pieces of paper if they had previously learned that a large template was rewarded, and smaller pieces if a small template was rewarded, we conducted a reversal size learning experiment. The procedure described for the first size learning stage was repeated, but now birds were rewarded for choosing pieces of the opposite size. Glaz and Joe were now rewarded for large pieces, Rodya – for small ones. The pieces were all the same colour (green).

### Second size tests

The procedure described for the first size test was repeated.

## Analysis

The pieces of paper manufactured by birds were numbered, scanned and converted to monochrome bitmap format. Then, using the QGIS program (Open Source Geospatial Foundation, USA), the scanned images were converted from raster to vector format, the contours of the pieces were selected, and their area was calculated.

Since only three subjects were tested, only within-subject data was statistically analysed. Statistical analyses were conducted in IBM SPSS Statistics 27. Graphs were built in GraphPad Prism. A two-sided binomial test was used for the Colour test. Normality and variance of the data within and between the crows were checked, which evidenced the need for non-parametric testing. Mann–Whitney U-tests were used to compare the mean areas of the pieces manufactured after training to select large or small pieces of paper.

## Results

### Pre-training

It took one (Glaz) to three (Rodya and Joe) weeks to habituate the birds to the experimental cage and train them to pick up stones from the tray and place them in the slit. Birds reached criterion (placed all 8 pieces into the slit in three consecutive trials) after 13 (Glaz), 25 (Rodya), and 84 (Joe) trials.

### Spontaneous manufacture test

**Fig. 2:**
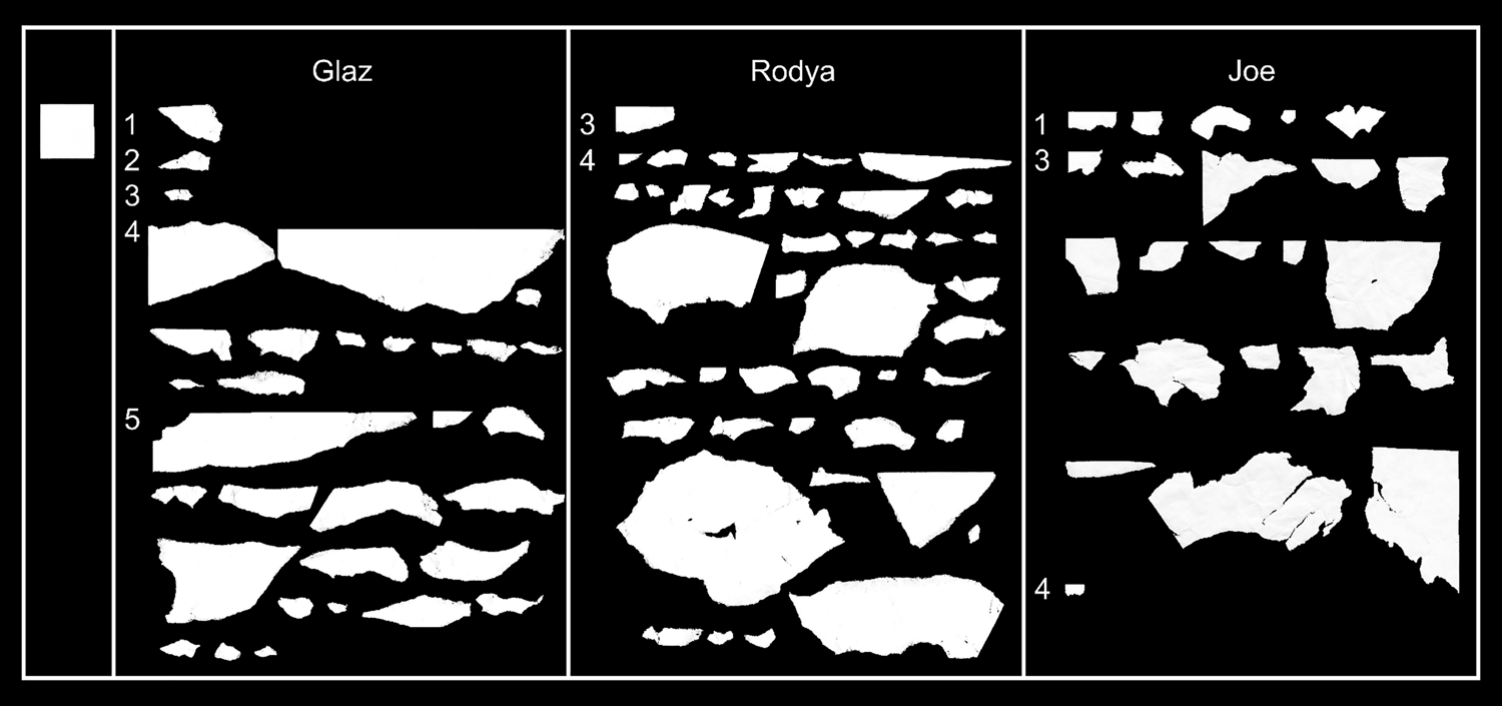
The pieces manufactured in the Spontaneous manufacture test of the three subjects. Example template provided on the top left. The numbers on the left indicate the days on which the pieces were manufactured

All three birds started manufacturing pieces and placing them in the slit spontaneously, without additional training (Fig. 2). Glaz tore off one piece of paper on each of the first three days (three 15-min tray presentations per day), and from the fourth day onwards produced 12 or more pieces per day. In total, he tore off and placed 32 pieces in the hole during 5 experimental days. Rodya tore off one piece of paper on the third experimental day and 43 pieces on the fourth experimental day. Joe tore off 5 pieces on the first day, then 23 on the third day, and one piece on the fourth day.

### Colour learning

Birds reached criterion (put all 4 pieces of the blue colour and no yellow pieces into the slit in three consecutive trials) after 57 (Glaz), 112 (Rodya), and 230 (Joe) trials.

### Colour test

**Fig. 3:**
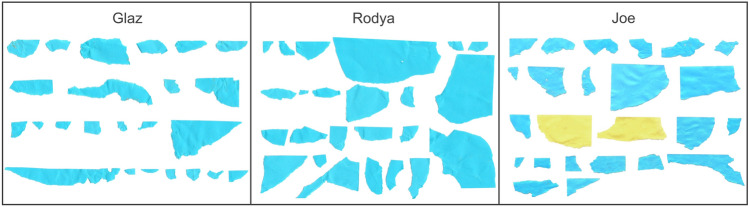
24 pieces manufactured in the Color test of the three subjects in the order in which they were produced

All birds, when presented with sheets of blue and yellow A4 paper, manufactured more pieces from previously rewarded blue colour (Fig. 3). Glaz and Rodya manufactured and placed in a slit only blue pieces, Joe–two yellow pieces and 22 blue ones (p < 0.0001, two-sided binomial test). All three birds manufactured their first piece from previously rewarded colour.

### Size learning

In the first Size learning, birds reached criterion (put all 4 pieces of the rewarded size and none of the other size into the slit in three consecutive trials) after 43 (Glaz), 84 (Rodya), and 133 (Joe) trials.

In the Reversal Size learning, birds reached this criterion after 37 (Glaz), 105 (Rodya), and 136 (Joe) trials.

### Size tests

**Fig. 4:**
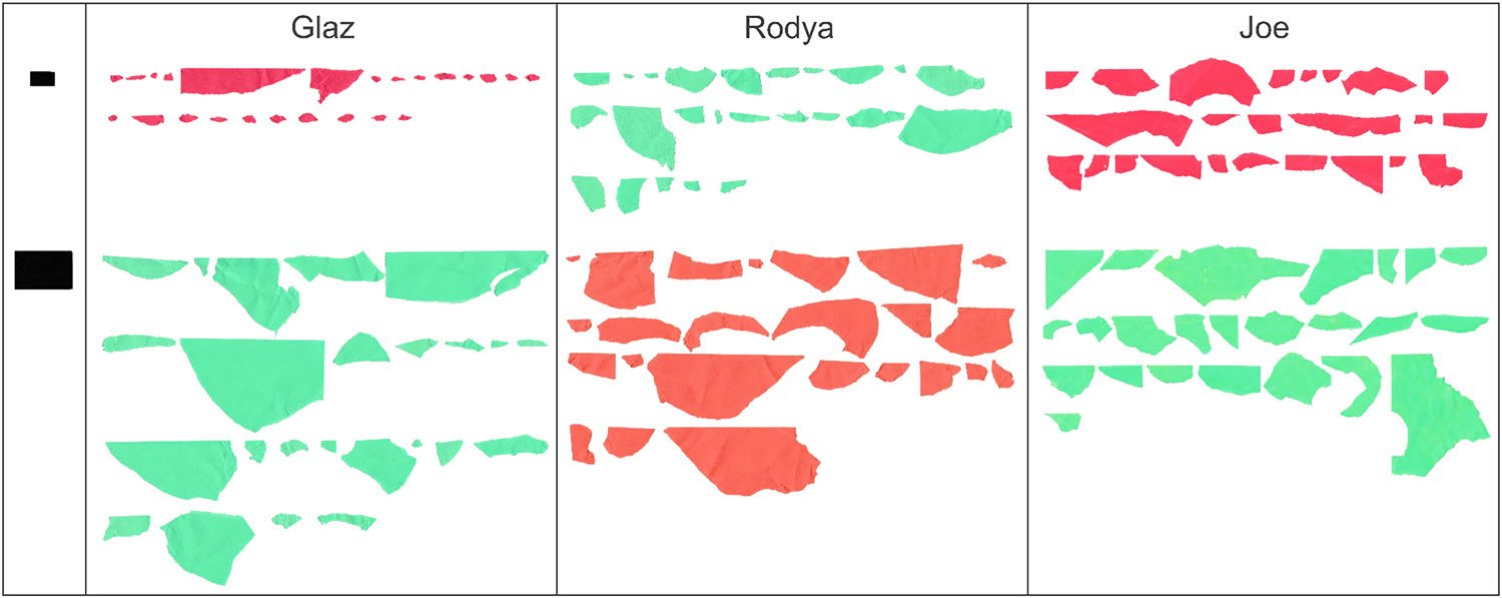
The pieces manufactured in the Size test by the three subjects in the order in which they were produced. The template sizes are provided on the left

**Fig. 5:**
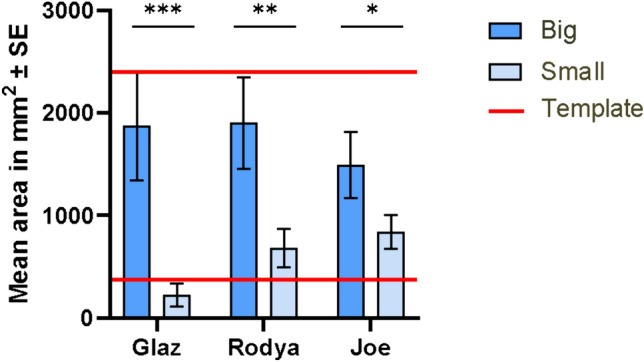
Mean area of pieces (± standard error) manufactured by birds in the Size test after learning to select large (dark blue bar) and small (light blue bar) pieces of paper. ***p < 0.001, **p < 0.01, *p < 0.05 (Mann–Whitney U-test)

All three hooded crows manufactured differently sized card pieces after learning that either large or small templates were rewarded (Fig. 5). The areas of pieces produced by all three birds after training to select larger or smaller prepared pieces were significantly different (p < 0.001 for Glaz, p = 0.004 for Rodya; p = 0.016 for Joe; Mann–Whitney U-test; Fig. 4 and Fig. 5). The exact sizes of the manufactured pieces is given in Online Resource 2.

For Glaz, the mean area of manufactured pieces was 8.3 times larger (mean_small_ = 225.65 ± 110.81 mm^2^; mean_big_ = 1874.58 ± 530.76 mm^2^) after rewarding for big pieces (mean_big template_ = 2400 mm^2^) than for small ones (mean_small template_ = 375 mm^2^). For Rodya, the mean area was 2.79 times larger (mean_small_ = 681.47 ± 187.82 mm^2^; mean_big_ = 1902 ± 445.86 mm^2^), and for Joe–1.78 times larger (means_mall_ = 838.8 ± 165.12 mm^2^; mean_big_ = 1489.95 ± 322.69 mm^2^).

For all three birds, after receiving rewards for small templates during training, the birds manufactured 41 pieces smaller than the small template (< 375 mm^2^), 26 pieces in between the templates (375–2500 mm^2^) and 5 bigger than the big template (> 2500 mm^2^). In contrast, after getting rewarded for big templates, they manufactured 15 pieces smaller than the small template (< 375 mm^2^), 43 pieces in between the templates (375–2500 mm^2^) and 14 bigger than the big template (> 2500 mm^2^).

Since no templates were present during the manufacturing trials, and birds were rewarded at random, the size of the manufactured pieces during the test phase could only have been influenced by the prior learning of which of the two differently sized templates was rewarded during the earlier object choice task.

During the test, all three birds on multiple occasions discarded a previously manufactured piece before subsequently tearing off and inserting a second one. None of the three crows additionally modified already detached pieces to reduce their size.

## Discussion

We found that Hooded crows, similar to tool-specialized NC crows (Jelbert et al. 2018) and non-tool-specialized Goffin’s cockatoos (Laumer et al. 2021), are able to manufacture objects according to a mental template. They spontaneously manufactured paper pieces that matched the colour and the relative size of previously rewarded, pre-made templates despite being rewarded at random and without templates being present at the time of manufacture.

When the Hooded crows did not receive ready-made pieces of paper in the Spontaneous manufacture test, all three birds began spontaneously (without training) tearing off pieces from large sheets, comparable to four of eight New Caledonian crows (Jelbert et al. 2018). Among the cockatoos, this particular behaviour was not observed. Here, two of the six cockatoos tested (Laumer et al. 2021) already had experience in carving strips out of cardboard in order to use them as stick tools (Auersperg et al. 2016). The remainder learnt to do so after observing card-ripping demonstrators, or when presented with semi-torn sheets.

Next, the Colour test was conducted to investigate whether the crows would spontaneously manufacture objects out of the same colour material as the previously rewarded template. To prevent learning during the test, the birds were randomly rewarded for 50% of the pieces, irrespective of the colour made, which differed from the two previous studies in which only items made from the correct colour were rewarded. Here, all three Hooded crows spontaneously manufactured pieces of the same colour as the previously rewarded template. They selected the correct colour to manufacture objects from the first trial onwards. Only 2 pieces were made from the previously unrewarded colour, both made by one crow (Joe). For comparison, five out of six New Caledonian crows that participated in the Colour test made at least 19 blue pieces out of 24 (Jelbert et al. 2018) and all six Goffin’s cockatoos manufactured at least 18 blue strips out of 24 (Laumer et al. 2021), with five Goffin’s selecting the correct colour from the first trial onwards. Thus, New Caledonian crows and Goffin’s cockatoos did not learn this through dozens of trials, but began manufacturing pieces of the right colour almost immediately.

Finally, in the Size test, all three Hooded crows manufactured larger objects when they had been previously rewarded for large templates, than when rewarded for small templates. This behaviour was demonstrated by six Goffin’s cockatoos (Laumer et al. 2021) and six adult New Caledonian crows, though not by two juvenile New Caledonian crows (Jelbert et al. 2018). As in earlier studies, there was no differential reinforcement that the Hooded crows could have used to guide their manufacture. Despite all three crows being successful in this task, the oldest crow Glaz showed a bigger difference between conditions than the youngest Joe. These individual differences could be attributed to a wide variety of reasons, such as age, motor control or motivation.

None of the three Hooded crows additionally modified already detached pieces to reduce their size. In contrast, in several trials, one New Caledonian crow made secondary modifications to reduce the size of large pieces (Jelbert et al. 2018). This crow manufactured pieces that were highly similar to each template. Together, this could suggest that New Caledonian crows remembered and reproduced the absolute, not just relative, size of the previously rewarded templates. Goffin’s cockatoos did not tear off pieces of paper but used the carving technique–each strip was carved with a large number of bite marks alongside the edge of the cardboard (Laumer et al. 2021). They therefore had more precise control over the final length of the cardboard strip, when compared to the accuracy of freely ripping a piece of paper. Notably, the most accurate Goffin’s cockatoo made paper strips that were highly similar to the short and long templates not only in relative but also in absolute length. The fact that our three crows did not make secondary modifications to reduce the size of the pieces in either condition can be one of the reasons for the high variability in the sizes of manufactured pieces. Other factors with potential influence could be random reward and the lack of total control over the size of the pieces the birds produced by tearing at the paper. However, the differences in size we observed across the two conditions occurred in spite of these factors.

In summary, all three bird species examined appear to be similarly capable of manufacturing physical objects in accordance with a mental template. They can reproduce different properties of the sample–qualitative (colour) and quantitative (size). Whether the cognitive abilities demonstrated by New Caledonian crows, Goffin’s cockatoos and Hooded crows are unique or are more phylogenetically widespread, is currently unknown. We hypothesise that this ability will also be found in other animals with a high level of brain and cognitive development, which can readily form and use representations. It's likely that if animals can form and use representations, they can use this ability flexibly in different situations–including manufacturing physical objects. For example, grey parrots also likely possess the ability to recreate a physical template from memory, as they are able to manipulate symbolic and visual working memory representations (Pepperberg 1987; 2013; 2018; 2020; Pailian et al. 2020).

It would be also interesting to find out whether preschool children can manufacture objects according to a mental template using this experimental approach. In children, tool-making is a slow and late-developing ability: for example, most children under 5 years rarely find a solution to a problem in which they have to make a hook to retrieve a bucket from a narrow vertical tube (Beck et al. 2011; Cutting et al. 2011, 2014; Chappell et al. 2013; Breyel and Pauen 2023). In contrast, certain corvids and parrots have demonstrated success in this task (Weir et al. 2002; Laumer et al. 2017; Bird and Emery 2009).

Children’s difficulty is surprising because they seem to have all the relevant information: they understand the properties of the materials they are given as well as the physics of the task and they can recognize a hook as the most functional tool (Beck et al. 2011; Cutting et al. 2011; 2014). It has been shown that children’s difficulty is in retrieving and coordinating their knowledge, which is needed to solve ill-structured tool-innovation problems (Cutting et al. 2014). Interestingly, even three-year-olds transfer the correct solution across analogous tool making tasks (Breyel and Pauen 2023). However, they mostly fail to implement the solution by correctly adjusting the tool. Their difficulties are probably due to a lack of fine motor skills and coordination of perceptual feedback (Breyel and Pauen 2023). Familiarity with tools also improves the tool making performance of 5 and 6-year-old children (Gönül et al. 2021).

Applying the approach used on birds, which is more formal than classical tool-making tasks, would allow us to find out whether children can retrieve and coordinate information about certain individual characteristics of manufactured objects.

## Supplementary Information

Below is the link to the electronic supplementary material.

Supplementary file1 (MP4 548853 KB)

Supplementary file2 (XLSX 12 KB)

## Data Availability

All data that support the findings of this study are included within this paper and its Supplementary Information files.
